# A granuloma Inguinale in an infant: a case report

**DOI:** 10.1093/omcr/omag031

**Published:** 2026-03-23

**Authors:** Reem Hasan, Nemat Alsaghir, Nour Alhoda Mousa Eysa, Aisha Alloush

**Affiliations:** Department of Dermatology and Venereology, Damascus University, Damascus, Syria; Department of Dermatology and Venereology, Damascus University, Damascus, Syria; Department of Dermatology and Venereology, Damascus University, Damascus, Syria; Children’s Hospital, Damascus University, Damascus, Syria

**Keywords:** donovanosis, granuloma inguinale, *Klebsiella granulomatis*, infant, case report

## Abstract

Granuloma inguinale (donovanosis) is a chronic granulomatous infection caused by *Klebsiella granulomatis*, predominantly affecting sexually active adults in endemic regions. Pediatric cases are exceedingly rare and pose diagnostic challenges, particularly in non-endemic settings. We report an unusual case of granuloma inguinale in an 8-month-old male infant from Syria who presented with multiple painless ulcerative lesions involving the genital and diaper areas. Diagnosis was established through identification of Donovan bodies on Giemsa stain and confirmed by histopathological examination. The patient showed an excellent clinical response to oral azithromycin, with complete resolution of lesions and no recurrence during follow-up. This case highlights the importance of considering granuloma inguinale in the differential diagnosis of chronic genital ulcers in infants and underscores the need for careful safeguarding assessment and the role of histopathology in achieving an accurate diagnosis.

## Introduction

Granuloma inguinale, also known as donovanosis, is a chronic, progressive granulomatous infection caused by the Gram-negative bacterium *K. granulomatis*. The disease predominantly involves the genital and perigenital regions and is typically transmitted through sexual contact. It is most commonly reported in tropical and subtropical regions and is strongly associated with low socioeconomic status and poor hygiene [1]. Clinically, granuloma inguinale classically presents as painless, slowly progressive ulcerative lesions with a characteristic beefy-red appearance and a tendency to bleed on contact. Several clinical variants have been described: ulcerogranulomatous (the most common), nodular, hypertrophic, sclerotic, and necrotic forms [2]. Although over 90% of cases involve the genital area, extragenital involvement has been reported [3]. The occurrence of granuloma inguinale in infants is exceptionally rare and raises important diagnostic and safeguarding considerations. Transmission in this age group is thought to occur through non-sexual routes, including close contact with infected caregivers or contaminated materials [4–6]. Herein, we present a rare case of granuloma inguinale in an 8-month-old infant from Syria, emphasizing the diagnostic challenges and therapeutic response.

## Case presentation

**Figure 1:**
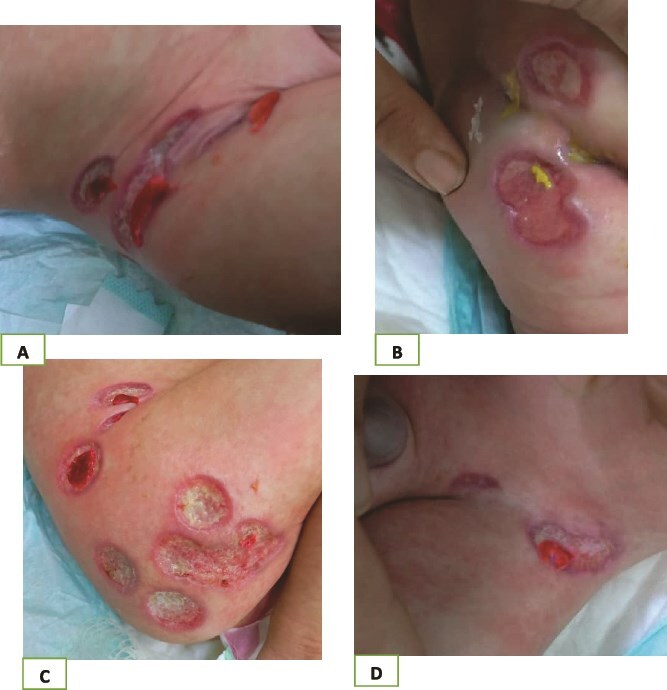
(A–D) Ulcers on the diaper area.

An 8-month-old boy presented to the Hospital of Dermatology in Syria with his mother, who complained that her son was suffering from multiple lesions on the genital area and buttocks. The infant was admitted to the neonatal intensive care unit at Damascus University Children’s Hospital due to high fever and diarrhea. His medical history showed that the patient had been drinking cow’s milk since he was only one month old. Later, he started to have diarrhea, then the lesions started, manifested as papules, which developed later to ulcers, as the mother said. On physical examination, the lesions were multiple non-painful deep round ulcers with knife-cut borders confined to the genital area and buttocks (Fig. 1).

At first, we put our patient on topical treatments (eosine, silver sulphadiazine, zinc oxide, candimazole, fusidate) with slight improvement. We made our differential diagnosis consisting of Donovanosis, Jacquet erosive diaper dermatitis, Pyoderma Gangrenosum, Factitious dermatitis by proxy, panniculitis and other infectious causes of genital ulceration such as chanroid and lymphogranuloma venerum, and performed the next tests:

Laboratory tastings revealed mild anemia (Hb 5.6 g/dl) and elevated CRP levels (CRP: 30 mg/l; upper limit of normal 10 mg/l), approximately three times the upper limit. Other laboratory parameters, including renal and liver function tests, were within normal limits.

**Figure 2:**
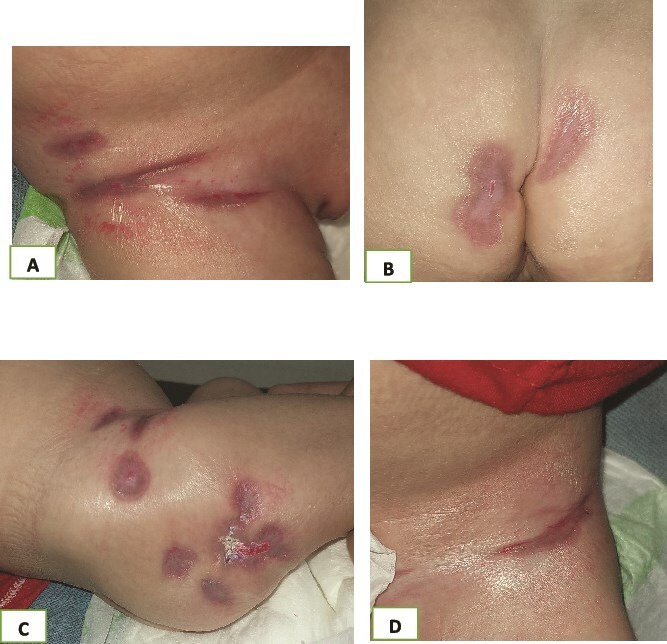
(A–D) Healing of the uclers after treatment.

Culture from the ulcer demonstrated the existence of Klebsiella bacteria; the culture results became available after approximately 7–10 days, contributing to a delay in definitive diagnosis. A Giemsa stain from the border of an ulcer showed Donovan bodies (Fig. 2).

**Figure 3:**
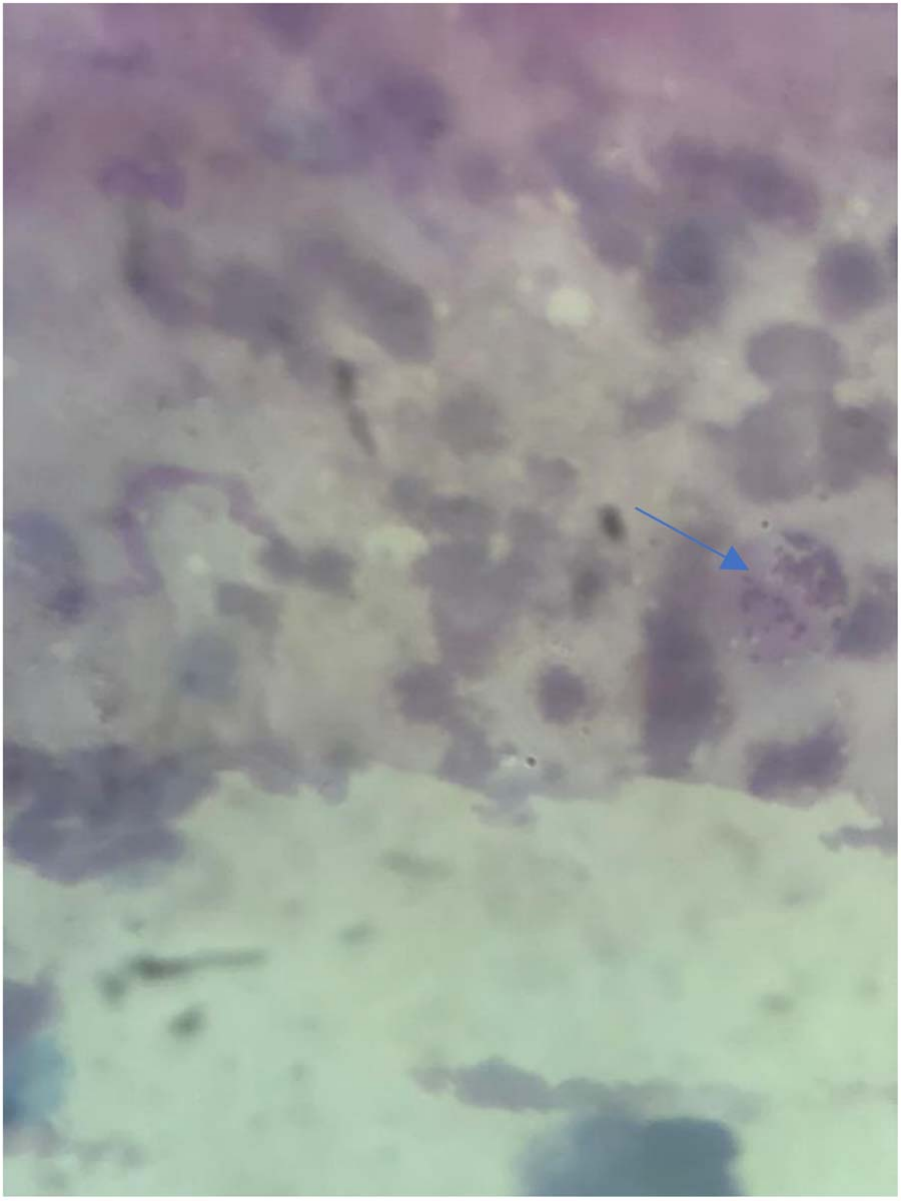
Giemsa stain showed Donovan bodies (blue arrow).

A skin biopsy showed epidermal hyperplasia, acanthosis, and a granulomatous infiltrate rich in histiocytes and neutrophils, with no vasculopathy (Fig. 3).

**Figure 4:**
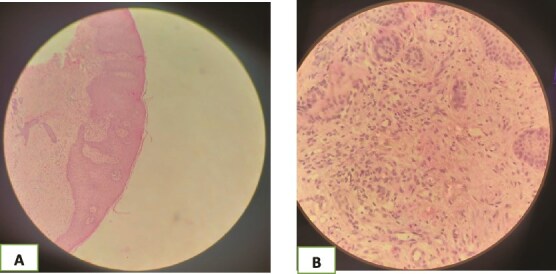
(A and B) Histological findings of the lesion; epidermal hyperplasia, acanthosis, and a granulomatous infiltrate rich in histiocytes and neutrophils, with no vasculopathy.

Based on these findings, a diagnosis of granuloma inguinale was established. The patient was started on oral azithromycin (20 mg/kg/day) for four weeks. Marked clinical improvement was observed within one week, and complete healing occurred after approximately one month (Fig. 4). No recurrence was observed during a 6-month follow-up period.

## Discussion

Granuloma inguinale is a rare cause of chronic genital ulceration and is predominantly encountered in sexually active adults. Pediatric cases, particularly in infants, are exceedingly uncommon and represent a diagnostic challenge due to the broad differential diagnosis of ulcerative lesions in the diaper area. The present case illustrates a classic clinical and histopathological presentation of granuloma inguinale in an atypical age group. The painless nature of the ulcers, their well-defined borders, and the presence of a granular base were key clinical clues. However, definitive diagnosis relied on the identification of Donovan bodies on Giemsa staining and the characteristic granulomatous infiltrate observed on histopathological examination.

## Safeguarding considerations

Given that granuloma inguinale is classified as a sexually transmitted infection, its diagnosis in an infant inevitably raises safeguarding considerations. In the present case, the possibility of sexual transmission was explicitly considered and addressed with sensitivity. A thorough history was obtained from the caregivers, and careful clinical assessment revealed no physical, behavioral, or social indicators suggestive of sexual abuse. The child was continuously accompanied by his mother during hospitalization, and no signs of trauma were identified. In keeping with existing literature, non-sexual transmission routes—such as close contact with an infected caregiver or exposure to contaminated fomites—were considered the most plausible mechanisms of acquisition in this age group.

Several conditions were considered in the differential diagnosis. Jacquet erosive diaper dermatitis may present with ulcerations in the setting of severe diarrhea; however, these lesions are typically superficial, painful, and respond rapidly to topical therapy, which was not observed in our patient [7]. Pyoderma gangrenosum, although characterized by rapidly progressive ulceration, is extremely rare in infancy and is usually associated with painful ulcers, undermined borders, and histological evidence of neutrophilic dermatosis with vasculopathy [8]. Factitious dermatitis by proxy was also excluded, as it generally presents with lesions of bizarre morphology.in the absence of preceding symptoms, and histopathology lacks the prominent neutrophilic and histiocytic infiltrate seen in this case [9]. Panniculitis was considered unlikely due to the absence of subcutaneous nodules and the lack of lobular or septal inflammation on histopathological examination [10].

The differential diagnosis also included other infectious causes of genital ulceration such as chancroid and lymphogranuloma venerum, although these were considered less likely given the patient’s age and clinical presentation.

Azithromycin is currently recommended as first-line therapy for granuloma inguinale and should be continued until complete healing of lesions is achieved. Our patient demonstrated a rapid and sustained response to azithromycin monotherapy, consistent with previous reports. Relapses have been documented up to 18 months after treatment, underscoring the importance of long-term follow-up [3].

## Conclusion

Granuloma inguinale, though rare in infancy, should be considered in the differential diagnosis of chronic, painless ulcerative lesions in the diaper area. Early recognition and confirmation through histopathological and microbiological examination are crucial to avoid misdiagnosis and inappropriate treatment. Prompt initiation of appropriate antibiotic therapy can result in excellent clinical outcomes.
